# PROTOCOL: Mental disorder, psychological problems and terrorist behaviour: A systematic review

**DOI:** 10.1002/cl2.1249

**Published:** 2022-06-10

**Authors:** Kiran M. Sarma, Sarah L. Carthy, Katie M. Cox

**Affiliations:** ^1^ School of Psychology National University of Ireland Galway (University of Galway) Galway Ireland

## Abstract

This is the protocol for a Campbell systematic review. The objectives are as follows: the first objective of the review (Objective 1—Prevalence) is to present a synthesis of the reported prevalence rates of mental health difficulties in terrorist samples. Where sufficient data is available, the synthesis will be sensitive to the heterogeneity of the terrorism phenomenon by exploring the rates of mental health difficulties for different forms of terrorism and for different terrorist roles (e.g., bombing, logistics, finance, etc.). The second objective (Objective 2—Temporality) will synthesise the extent to which mental health difficulties pre‐date involvement in terrorism within prevalence studies. Finally, the third objective (Objective 3—Risk) aims to further establish temporality by examining the extent to which the presence of mental disorder is associated with terrorist involvement by comparing terrorist and non‐terrorist samples.

## 1 BACKGROUND

### 1.1 The problem, condition or issue

In the period 1970–1990 there was extensive debate on the potential link between mental disorder and terrorism (Cooper, 1978; Ferracuti & Bruno, 1981; McCauley & Segal, 1987; Rasch, 1979; Shaw, 1986; Silke, 1998; Smith & Morgan, 1994; Wardlaw, 1982). Commentators proposed that some of those who became involved in terrorism had an underlying mental disorder that was causally linked to their violence propensity, including, for example, a range of personality disorders (Cooper, 1978; Lasch, 1979; Pearce, 1977). However, successive studies failed to support this link (e.g., Elliot & Lockhart, 1980; Ferracuti & Bruno, 1981; Lyons & Harbison, 1986) leaving many convinced that those involved in groups like the Provisional IRA and Euskadi Ta Askatasuna (ETA) were essentially normal individuals attracted to terrorism by virtue of a multitude of interacting psychosocial processes (e.g., Crenshaw, 1981; Heskin, 1984; Taylor & Horgan, 2006). The assertion that terrorism was the product of abnormality was ultimately deemed ‘unfair’ to the terrorist and abandoned by researchers and policy makers (e.g., Silke, 1998).

In recent times the debate has re‐emerged. Those interested in so‐called lone actor violence raised concerns about ‘fundamental errors’ in past research (Corner et al., 2016; p. 561) and presented plausible case formulations linking experiential stressors such as social isolation with mental disorder and violence (e.g., Corner & Gill, 2015). Cohort studies of lone actors emerged that appeared to show high rates of mental disorder, with 37% of Liem and colleagues' sample of European lone actor terrorists having ‘some indication of mental illness’, and 25% being clinically diagnosed with ‘a particular mental disorder’ (Liem et al., 2018; p. 60). Such findings were not limited to lone‐actor terrorism (e.g., Weenink, 2015).

However, those who have looked more closely at this evidence base have acknowledged that the picture emerging is far from clear, with the lack of clarity attributable, in part at least, to methodological limitations in that literature (Gill et al., 2021; Jensen et al., 2020). To some extent these limitations centre on one core problem—the difficulty determining to what extent, if any, the presence of a mental disorder *confers* risk of terrorist involvement (as opposed to being associated with increased risk). We suggest that the existing literature needs to demonstrate that a number of criteria are met to credibly conclude that disorder, or more broadly psychological difficulties, can increase the risk of becoming involved in terrorism.

The first relates to the prevalence of disorder among terrorist samples, and is termed here ‘the prevalence problem’. Assessing prevalence of mental disorder among terrorist samples using appropriate comparators sheds light on the magnitude of the relationship (if any) between the risk and outcome. It is an important criterion for determining causality and one of several Bradford‐Hill criteria for causation (Hill, 1965). Reporting and interpretating prevalence rates requires careful consideration of the distinction between point and period prevalence, and use of appropriate comparison groups. If mental disorder confers risk of terrorism, then the prevalence rate amongst terrorist samples should be higher than that expected in the general population (matched on key demographics such as age, gender and geographic location). A prerequisite for making such comparisons is synthesising the prevalence rates of mental disorders, and more broadly psychological difficulties, in terrorist samples.

The second criterion is that of *temporality* (see Lucas & McMichael, 2005), another Bradford‐Hill criteria (and termed here ‘the temporality problem’). To demonstrate that mental disorder confers risk of involvement in terrorism, then studies must demonstrate that the disorder emerges before involvement in terrorism. If we cannot demonstrate temporality, then differentiating cause and effect is problematic—disorder could be the consequence of terrorist involvement rather than a cause of involvement. The question that must be asked of the literature, then, is: To what extent do findings in the literature support the assertion that earlier mental disorder confers risk of terrorist involvement later in life?

The third criterion that must be met if we are to be confident that a causal link exists between mental disorder and terrorism is that plausible explanations for just how the disorder (or disorders) confers risk of terrorism involvement must be proffered (the Bradford‐Hill ‘plausibility problem’). Addressing the plausibility problem is hampered by the heterogenous nature of terrorism (the outcome of interest), controversy around the concept of ‘mental disorder' (the potential risk factor of interest) and, finally, the complexity of the relationship that may exist between terrorism and mental health difficulties.

A final consideration for those conducting research on terrorism and mental disorder relates to the concept of ‘mental disorder’ itself. To illustrate the difficulty with the concept it is worth referring to the recent contribution of Bakker (2019). Bakker's thesis, like many other clinical psychologists, is that the concept of ‘mental disorder’ is fundamentally flawed, a ‘medical nosology of diseases’ that does little to aid our understanding of clients or how best to intervene to alleviate their distress (p. 1). What is required, he argues, is a paradigm shift towards a focus on ‘psychological problems’ that are transdiagnostic (appear across diagnoses and capture the common difficulties reported by patients) and that may or may not require intervention. His thesis draws attention to two types of mental health difficulties—mental disorders and psychological problems.

The difficulty for the terrorism literature is that it is not always clear what the outcome of interest is, and terms like disorder, mental health difficulties, psychological distress, etc., are used interchangeably. Yet, where a formal diagnosis is not made by a mental health professional, then the presence of a mental disorder cannot be inferred. At best we can conclude that the individual has or had what Bakker refers to as a psychological problem.

This review seeks to contribute to our understanding of the potential link between terrorism and mental health difficulties by focusing on the problems of prevalence, temporality and plausibility, and while being sensitive to the distinction between mental disorder and psychological problems. Specifically, the review will present a synthesis of the evidence on prevalence, temporality and plausibility drawing on the best evidence available. In reviewing prevalence, temporality and plausibility we are presenting an initial test of what we refer to as the mental health—terrorism hypothesis: That mental health difficulties confer risk of involvement in terrorist behaviour.

### 1.2 Outcome

The outcome of interest in this systematic review is terrorist behaviour. While there is no universally accepted definition of terrorist behaviour (Ganor, 2002; Saul, 2019; Silke, 1996), there is at least some consensus that it refers to: (a) an act or campaign of actual or threatened violence that seeks to elicit the terror emotion in a target audience and; (b) with the intention of bringing about change in line with the world‐view of the terrorist (e.g., Kruglanski & Fishman, 2006; Moghaddam, 2007). Terrorist behaviour intends to cause harm, physical or otherwise (Van Der Does et al., 2021).

One complexity encountered by those conducting research in the area of terrorism is the heterogeneity of the phenomenon itself (Herrington & Roberts, 2012; Monahan, 2012; Roberts & Horgan, 2008). For example, one study of 176 terrorist organisations identified 33 different ideologies as well as diversity in terms of size, organisational structure, geographic location and lethality (Cook & Lounsbery, 2011). It cannot be assumed that individuals who become involved in different forms of terrorism do so through the same processes (Change Institute, 2008). The implications of this heterogeneity for the proposed review are two‐fold. First, when we refer to terrorism, we must specify this behaviour both in terms of the ‘type’ of terrorism waged as well as the ‘roles’ of terrorists (Perliger et al., 2016). Second, when reviewing the works of others, we must be sensitive to the specific behaviours they are investigating (e.g., suicide attacks vs. financing terrorism vs. mass shootings) in reporting of findings.

The review excludes violent radicalisation as an outcome, understood within this study to refer to a process of growing acceptance of the legitimacy of violence to bring about societal change (McCormick, 2003). We justify this exclusion on the following basis:

1. Violent radicalisation and terrorism are conceptually non‐synonymous. Terrorist behaviour is *action* orientated whilst violent radicalisation is a process whereby individuals become increasingly committed to the use of violence, yet may not necessarily perpetrate violence (i.e., it can be cognitively orientated) (Sarma, 2017).
2. Of the significant minority of those who accept the legitimacy of terrorist violence (e.g., PEW, 2013), a small proportion transition into terrorism (Christmann, 2012). Those who transition into terrorism may differ from those who do not on both dispositional (e.g., morality, aptitude, motivation, etc.) or situational (e.g., opportunity) levels. If so, the findings from the literature on violent radicalisation will lack external validity when applied to terrorist behaviour.

### 1.3 Predictive factor(s)

The review seeks to inform our understanding of the potential link between mental disorder and terrorist involvement. However, we are acutely aware of the limitations of the largely disease‐orientated focus of ‘mental disorders’, and for the reasons set out below extend our focus to include a more transdiagnostic lens by considering, broadly, psychological problems.

The American Psychological Association defines mental disorder as any condition ‘characterized by cognitive and emotional disturbances, abnormal behaviours, impaired functioning, or any combination of these’ (American Psychological Association, 2020). Diagnostic manuals, such as iterations of the Diagnostic and Statistical Manual of Mental Disorders (DSM; American Psychiatric Association, 2013) and International Classification of Diseases (ICD; World Health Organization [WHO], 2019), present diagnostic criteria for a range of mental disorders, and diagnoses can be made based on the combination of symptoms and their severity.

While diagnostic systems convey a sense that mental disorders are discrete psychological experiences, Allsopp and colleagues note that diagnostic systems are characterised by: (a) varying diagnostic rules across presentations; (b) overlap in symptoms across diagnoses and; (c) a tendency for diagnostic labels to mask the root causes of distress and problematic behaviours (Allsopp et al., 2019). They also argue that diagnoses can distract from the real‐world work of reaching an in‐depth understanding of the individual (see also Galatzer‐Levy & Bryant, 2013; Olbert et al., 2014).

The use of diagnostic systems in terrorism research is particularly problematic. Mental disorders can only be said to be present where they have been diagnosed by an appropriately trained mental health professional. The presence of a disorder, as characterised in DSM or ICD, cannot be reliably inferred from reports of symptoms present in open source data alone (e.g., press coverage of trials of suspected terrorist offenders). It requires careful assessment by a professional, often in collaboration with the individual being assessed, of the presence or absence of various criteria. Where the results of such assessment are made available for the purposes of research, then we can have at least some confidence in the diagnostic process.

However, the data on terrorist offenders does not always provide access to data gathered through structured clinical assessment by a suitably trained professional. Some of the most widely cited studies in the area, for example, are based on open source information derived from the media coverage, etc., of terrorists, and where inferences are made as to the presence/absence of disorder based on difficulties experienced by the terrorist and publicly reported (e.g., Gill et al., 2014). Yet it is not always clear if these difficulties are any different to those experienced in the normal course of one's life.

Bakker (2019) has discussed this in the context of differentiating between normal ‘psychological problems’ that ‘do not self‐perpetuate… and tend to ease without interventive therapy’ (p. 11) and ‘clinical psychological problems’. The former, Bakker argues, are normal adaptive processes that might include an experience of depression after a bereavement, but which follows the normal course of recovery. The latter, however, might take the form of avoidance, be pervasive and enduring, impair one's quality of life, and require intervention to resolve.

In the review we will attend to the literature on mental disorder and psychological problems, and where possible differentiate between psychological problems that are ‘clinical’ (required intervention) and those that were not clearly clinical (no evidence of a requirement of intervention). Synthesising the literature on ‘mental disorder' provides coverage of the diagnostic literature. Attending to the broader literature on psychological problems may capture a wider body of literature and mean that our review is not constrained by the limitations of the psychiatric model (see ‘Inclusion Criteria’ for additional detail).

### 1.4 How mental disorder and psychological problems may be linked to terrorist behaviour

The link between mental disorder and forensic risk has been the subject of research for decades. Findings are unclear and inconsistent, with some studies appearing to link disorder to violence risk, while others have reported no link (e.g., Augestad Knudsen, 2020; Bhui & Jones, 2017). There appears to be three core complexities in this area that are directly relevant to an assessment of the link between terrorism and disorder.

The first is to do with the prevalence problem, as discussed earlier in this protocol, and which requires an assessment of the rates of mental disorders, and more broadly psychological difficulties, in terrorist samples. The second is *temporality*, initially discussed by Bradford Hill in his consideration of association and causality (i.e., one of the Bradford Hill criteria for causal inferences; see Lucas & McMichael, 2005). To argue that mental disorder confers risk of violence, research must demonstrate that the onset of psychological difficulties (Factor A) pre‐dates involvement in violence (Factor B). If temporality cannot be established, there is a risk of misinterpreting correlation (where Factor A is associated with Factor B) as causation (Factor A *causes* Factor B).

For example, high rates of mental disorder are often observed among incarcerated violent offenders (O'Sullivan et al., 2018). A typical study examining the link between mental disorder and violent offending in this population will involve those incarcerated completing a battery of measures that assess psychological wellbeing and severity of offence (‘index offence’). Where a relationship emerges, the temptation is to conclude that higher levels of psychological distress confers risk of serious offending (Factor A causes Factor B). The problem here, however, is that the research design deployed cannot test causal relationships and is limited to measuring associations. Because we do not have longitudinal data showing levels of psychological symptoms before involvement in violence, we cannot exclude the possibility that the distress arose due to the index offence, or during incarceration for that offence. In such a case, incarceration/offending (Factor B) could cause distress (Factor A). This resonates with the conceptual difficulties surrounding the suggestion that pre‐existing Posttraumatic Stress Disorder (PTSD) increases risk of terrorist involvement among so‐called ‘foreign fighters’ and where PTSD is assessed after they have returned from conflict (e.g., Al‐Attar, 2019).

Even where temporality is determined, we also need to consider the ‘Third Variable Problem’. Here an apparent causal relationship between disorder and violence may be explained by a lurking third variable (Variable X) that influences both factors and causes them to co‐vary. This might arise, for example, where experiences of discrimination and isolation drive both mental disorder and violent radicalisation. Here the apparent relationship between mental disorder and violent radicalisation may be wholly attributable to situational stressors and intervening to manage disorder may not reduce risk.

In reality, there is unlikely to be a large body of scientifically robust longitudinal evidence that addresses both temporality and the ‘Third Variable Problem’. This is particularly the case in the area of terrorism studies where the problem of concern is so difficult to expose to academic enquiry due to its low base rate in the population (Sarma, 2017). In such a situation, another of Hill's criteria becomes important—that of *plausibility*. That is, in assessing the relationship between mental disorder and violence risk, we must be able to present a plausible theoretical argument as to just how disorder confers risk. In clinical forensic practice, plausibility is addressed through careful assessment of forensic risk and the presenting of a theoretical argument (or formulation) that explains how the presence of risk factors may confer risk (Davies et al., 2013).

One way of presenting a formulation of risk is through the ‘4Ps’ Framework. The 4Ps Framework places a risk factor in a temporal space or chronology and proposes the nature of the relationship between the factor, other factors, and the outcome of interest. In doing so it differentiates between predisposing, precipitating, protective and perpetuating factors. It is widely used in both clinical and forensic psychology case formulation (e.g., Macneil et al., 2012).

The 4Ps framework can encourage a more nuanced consideration of the link(s) between terrorism and mental disorder. A predisposing risk factor is one that places the individual at risk of becoming involved in terrorism later in life. In the broader literature on clinical and clinical forensic psychology mental disorder is viewed as primarily a non‐causative background predisposition for becoming involved in violent behaviour and which is part of a complex set of interacting risk factors that together lead to a scaffolding of risk (e.g., Van Dorn et al., 2012). For example, Markowitz (2011) adopts a Social Disorganisation Theory approach to formulating risk in proposing that people with long‐term mental disorders are more likely to reside in disadvantaged communities characterised by socially disorganised neighbourhoods with a lack of health care, limited job opportunities, racial diversity and fragmented families. Crime flourishes in such criminogenic contexts, he argues, because there is a culture of acceptance of violent crime and poor social control over offending. This resonates with the terrorism literature, with some arguing that vulnerability to violent radicalisation is due, in part, to radicalising settings where some sections of the community endorse beliefs that justify terrorism (e.g., Schils & Pauwels, 2016).

A precipitating risk factor is one that apparently triggers a ‘crisis’. In the mental health literature, extreme situational stressors such as relationship break‐down, bereavement or other acute trauma can result in a cascade of events leading to the negative outcome (e.g., Barber et al., 2014). For mental disorder to precipitate involvement in terrorism, research would need to demonstrate that involvement in terrorism was immediately preceded by the onset of an episode of psychological distress that causally led to involvement in violence. In the broader clinical forensic literature, this has typically been associated with the presence of ‘positive' psychotic symptoms, such as irrational (delusional) beliefs about others who subsequently become the target of violent intent (Markowitz, 2011). Of course, it could be reasonably argued that in some cases where terrorist behaviour is precipitated by a mental disorder, and derives from disorder (e.g., persecutory beliefs), that the issue of concern is clinical forensic risk rather than terrorism.

Some forms of mental disorder may actually preclude an individual from becoming involved in terrorism (i.e., it is a ‘protective factor’). For example, there is evidence that organisations like the Provisional IRA sought to recruit the most psychologically robust individuals into their ranks as a way of reducing the potential for members to be compromised and turn informer, or to provide information while being questioned by the police and security services (Sarma, 2005). Here mental disorder actually protects against involvement.

Finally, perpetuating factors, in the context of terrorism, serve to maintain the problematic behaviour, and thus hamper the ability of the individual to disengage from terrorism. In their review of push and pull factors that influence the ability of extremists to disengage from terrorism, Jensen and colleagues noted that increased social mobility, onset of new intimate romantic relationships, children, and access to rehabilitation services can support disengagement (Jensen et al., 2020). Conversely, mental health difficulties can impede the ability of individuals to develop relationships, access services, and be more socially mobile, hampering disengagement. They conclude that ‘[e]specially when co‐occurring with substance abuse, mental illness can act as a strong barrier to disengagement, especially if it counteracts the feelings of disillusionment that otherwise may prompt one's exit’ (Jensen et al., 2020; p. 8).

### 1.5 Why it is important to do the review

As noted earlier, findings in the literature examining the link between terrorism and mental disorder are inconsistent (Ho et al., 2019). Where such inconsistencies are a feature of an evidence base, the cherry‐picking of results to suit a specific position can impede a nuanced understanding. Systematic reviews provide a synthesis of the available literature in one accessible paper and in doing so reduce bias (White & Waddington, 2012).

Gill and colleagues provide a useful review of the literature exploring the link between mental disorder and terrorism (Gill et al., 2021). They note the heterogeneity in prevalence rates in the literature and provide some plausible explanations for this heterogeneity. Our review, however, will attend in particular to point and period prevalence rates and include studies published since 2020. We will also present comprehensive data syntheses for both mental disorder and psychological problems and where appropriate present sub‐group analyses. We will critically evaluate the appropriateness of the comparator populations being used as benchmarks for prevalence. This can help us more carefully consider the causal link, if any, between disorder/difficulties and terrorist behaviour.

We also considered Misiak and colleagues’ systematic review of the link between mental health and radicalisation and mass violence (Misiak et al., 2019). While the review also presents a valuable contribution to the literature, it focuses primarily on the risk of radicalisation, with 9 of the 12 studies included in their review using community samples and self‐reported radical beliefs (i.e., not terrorist samples). As noted above, we cannot assume that the literature on violent radical beliefs is valid for our understanding of terrorist behaviour.

Findings from the current systematic review will support a more informed debate on the link between mental health difficulties and terrorist behaviour. For each paper included in the review, we will isolate and specify the prevalence being reported (e.g., present at time of assessment (point); childhood, etc.). We will also synthesise studies that are sensitive to temporal sequencing (temporality), where difficulties are onset before involvement in terrorism, and studies that examine the extent to which difficulties are associated with involvement (i.e., compare rates across two groups).

Apart from supporting debate in the area, the findings will be of value to a range of professionals who are responsible for risk assessment, risk mitigation and psychological intervention. In relation to risk assessment, for example, two popular risk assessment tools, the VERA 2R (Pressman et al., 2016) and ERG 22+ (Lloyd & Dean, 2015), both contain items relating to psychopathology despite concerns that (a) the evidence supporting their inclusion is contested and (b) there is a need to disaggregate disorders into various forms or problem clusters to determine which, if any, may be linked to risk of terrorist behaviour (see Herzog‐Evans, 2018). In supporting a more nuanced understanding of the link between disorder, psychological problems and terrorist behaviour our review will help guide the use of such tools and in doing so support decision making around psychological support, appropriate detention settings and release from detention.

In relation to psychological intervention, there are multi‐disciplinary teams working in most countries tasked with supporting individuals who may at risk of transitioning into terrorism to redirect their lives towards non‐violence. In many cases these teams include health workers who are sensitive to psychological problems that are believed to exacerbate risk of becoming involved in terrorism—teams comprised of professionals who would benefit from a systematic review of the relevant literature. The work of such teams has been reviewed and discussed elsewhere (see Sarma, 2018, 2019a, 2019b).

## 2 OBJECTIVES

The first objective of the review (Objective 1—Prevalence) is to present a synthesis of the reported prevalence rates of mental health difficulties in terrorist samples. Where sufficient data is available, the synthesis will be sensitive to the heterogeneity of the terrorism phenomenon by exploring the rates of mental health difficulties for different forms of terrorism and for different terrorist roles (e.g., bombing, logistics, finance, etc.). The second objective (Objective 2—Temporality) will synthesise the extent to which mental health difficulties pre‐date involvement in terrorism within prevalence studies. Finally, the third objective (Objective 3—Risk) aims to further establish temporality by examining the extent to which the presence of mental disorder is associated with terrorist involvement by comparing terrorist and non‐terrorist samples.

## 3 METHODS

### 3.1 Criteria for considering studies for this review

#### 3.1.1 Types of studies

The types of studies included for each of our review objectives are detailed below.

##### 3.1.1.1 Objective 1: Prevalence

Prevalence rates of mental disorder and psychological difficulties will be drawn from the following types of studies:

1. Cross‐sectional studies reporting the prevalence of mental health difficulties as they exist in the population of interest (i.e., terrorist samples) at a particular time.
2. Cohort studies, both prospective and retrospective. In the prospective cohort study design, a cohort of individuals will have been identified and followed‐up over time to determine who did and did not become involved in terrorism. Within this analysis, the presence of mental health difficulties in the cohort will have been recorded at the initial screen. In a retrospective cohort study, the past incidence of disorder or problems in a group of individuals who became involved in terrorism will have been evaluated posthoc.
3. Case‐control studies in which individuals from the population of interest (i.e., those who engaged in terrorist behaviour) are compared to a group who have not perpetrated the behaviour (i.e., ‘controls’) and then concurrently (at time of study) or retrospectively assessed for mental health difficulties. The groups will have been compared with respect to the prevalence of mental health difficulties. For these studies, we will extract data from the terrorist subgroup to estimate prevalence.

To appropriately assess whether the prevalence of mental disorder and/or psychological difficulties are higher or lower in terrorist samples, we will compare the prevalence rates of mental disorders and psychological difficulties to rates for the general population reported in national/global mental health estimates. Our approach for this is detailed in the ‘Data Synthesis’ section.

##### 3.1.1.2 Objective 2: Temporality

If a study included under Objective 1 also presents data where inferences can be drawn as to the temporal onset of difficulties relative to involvement in terrorism, and where difficulties pre‐dates involvement, it will be included under Objective 2 (e.g., Bakker et al., 2006). While these types of studies cannot definitively establish that mental health difficulties precede terrorist behaviour, they may provide preliminary indicators of temporality in the absence of more rigorous risk/predictive studies.

##### 3.1.1.3 Objective 3: Risk factor

Objective 3 involves synthesising studies where there is variability in terrorist behaviour (i.e., some individuals engaged in terrorist behaviour and others individuals did not) and variability in mental health issues. We will include prospective or retrospective cohort study designs or case‐control/cross‐sectional designs. Where inferences can be drawn about the temporal onset of the disorder relative to involvement in terrorism (i.e., if the problem or diagnosed disorder pre‐dates involvement in terrorism), this will augment our understanding of temporality. Where such inferences cannot be drawn, then studies will still inform our understanding of relative risk.

For all objectives we will exclude qualitative papers from the synthesis. However, where we identify qualitative papers that explore the mental health of those involved in terrorism, we will retain these in a separate folder in the bibliographic database and use this evidence to aid the interpretation and contextualisation the findings from the review. Similarly, we will exclude studies using a case study design (e.g., Faccini & Allely Clare, 2017), but draw on this literature for context.

To be included, studies must report details of the approach to data collection and the sampling strategy. Papers that report such detail, and align with our other inclusion and exclusion criteria, will be included. This includes those published in journal articles, book chapters, books, conference presentations, conference publications and unpublished reports.

All papers must report the results of an empirical investigation. We will exclude discussion papers, theoretical contributions, newspaper articles, blogs and any paper that does not detail a sampling strategy, approach to data collection, or empirical findings. We will exclude literature reviews and systematic reviews from the synthesis, but will retain such papers for the purpose of reverse searching for relevant publications (i.e., to harvest potentially relevant papers).

#### 3.1.2 Types of participants

For all three objectives, we will include studies that contain at least some participants who are, or have been, involved in terrorist behaviour. As widely acknowledged in the literature, there have been different conceptualisations of terrorism and terrorist behaviour across studies and this has been identified as one of the primary impediments to primary research, synthesis and generalisability (e.g., McCann, 2020; Perliger et al., 2016). As a synthesis of the primary literature, the proposed review cannot overcome this limitation. However, it is critically important that the synthesis is sensitive to it. To that end, the review will adopt the following approach:

1. We will consider the process of being involved in terrorism as commencing when the individual *acts* to become involved. For example, an individual who attempts to travel abroad to become involved in terrorism (e.g., by booking flights), but is prevented from doing so by the authorities, meets this conceptualisation of terrorist behaviour (e.g., Weenink, 2015). However, someone who expresses an intention to travel abroad but has not taken to steps to travel, has not acted and thus is not conceptualised here as being involved in terrorist behaviour.
2. Participants in the studies included will meet at least one of the following criteria: (a) been convicted of a terrorist offence; (b) died in the perpetration or attempted perpetration of an attack; (c) been identified by the authorities as having been involved in terrorist behaviour or attempting to become involved (e.g., attempting to travel to join a terrorist organisation); (d) self‐report as being members/former members of a terrorist movement. We acknowledge that there are problems with these parameters, including that what constitutes a terrorist offence can vary from one jurisdiction to another (and even within jurisdictions; Schmid, 2004). We also acknowledge that terrorist behaviour is diverse, and may include the perpetration of violence, but also many other actions in support of terrorism (Altier et al., 2013). These may include the design and dissemination of propaganda, financing terrorism, recruitment, logistics and training.
3. Definitions of terrorism and terrorist behaviour will be extracted from the papers, as will the forms of terrorism being studied and roles of participants, and we will be sensitive to this complexity in our aggregation (or disaggregation) and synthesis of the literature.
4. Participants can be of any age, gender or ethnicity.

Some studies may include a blend of participants, for example with some participants having become involved in terrorism, others who are going through the process of radicalisation, and others still who are completely uninvolved in terrorism or radicalisation. Where we can isolate a sample that has become involved in terrorism, then we will do so and include this sample and associated data in our review. We will do this based on data provided within each study. Where the information provided in the study does not enable us to distil out a sample involved in terrorism, we will contact the original investigators, initially reaching out to the corresponding author and subsequently to other named authors if necessary (i.e., if the corresponding author does not respond). Where we cannot isolate this sample (either based on the study or in consultation with the original investigators), then the study will be excluded from the review.

#### 3.1.3 Types of outcome measures

For all three objectives the ‘measurement’ of terrorist involvement may include studies where participants are identified through:

1. Self‐report—Participants report that they are or were involved in terrorism.
2. Official sources—For example, law enforcement or security services identify participants who are involved (e.g., Weenink, 2015).
3. Conviction – Participants are convicted of terrorist offences, potentially giving researchers an opportunity to conduct research during incarceration (e.g., Merari et al., 2009) or through a retrospective review of their lives (e.g., through open‐source research; e.g., Liem et al., 2018).

#### 3.1.4 Predictive/risk factor: Mental disorders and psychological problems

For all three objectives, the predictive/risk factor of interest is mental disorder or psychological problems, collectively referred to here as ‘mental health difficulties’. Mental disorders are typically diagnosed by mental health professionals, such as psychiatrists and psychologists, following careful clinical assessment (e.g., structured clinical interviews) and formulation. It may also be diagnosed, for research purposes at least, through psychological testing, with either formal diagnostic tests (e.g., Millon Clinical Multiaxial Inventory III) or established screening tools with established clinical cut‐offs. In our review, our search terms relating to mental disorder will be based on the core categories of disorder listed in the DSM (version III (1980)—V (2013) and ICD (v. 10 & 11.; WHO, 1992, 2019), as informed by an earlier review (Hossain et al., 2020), and listed in Table 1.

**Table 1:** Terms sensitive to disorders listed in DSM and ICD

Disorders		
ADHD	Depression	Obsessive
Alcohol	Depressive	Posttraumatic Stress
Anorexia	Dissociative	PTSD
Antisocial	Drug	Schizophrenia
Anxiety	Dysphoria	Schizotypal
ASD	Eating	Panic
Attachment	Intellectual	Personality
Attention	Insomnia	Phobia
Autism	Learning	Psychotic
Bipolar	Motor	Trauma
Cognitive	Neurocognitive	Schizoaffective
Communication	Neurodevelopmental	Sleep
Compulsive	Oppositional	Somatic
Conduct	OCD	Stress
		Substance

For psychological problems, we do not envisage setting a threshold of distress above which psychological problems will be deemed ‘present’. Rather, we will draw on the definitions of psychological problem and clinical psychological problems to add nuance to our synthesis, and based on the work of Bakker (2019). A psychological problem is a ‘negative psychological‐level state of affairs’ that can be problematic, yet be an adaptive healthy response to a given context or experience. It is a problem that resolves itself without intervention. A clinical psychological problem, conversely, does not resolve itself without intervention. For example, Bakker differentiates between a ‘grief reaction’ (a psychological problem that remits) and ‘depression' (which may be an enduring grief reaction), and between acute stress reactions (resolves) and Post‐Traumatic Stress Disorder (endures).

Unfortunately there is no established taxonomy of psychological problems available. Bakker (2019) makes some suggestions as to the structure, and indicative content, of a taxonomy but does not present one. An alternative taxonomy, Hopewood et al.'s Hierarchical Taxonomy of Psychopathology, was considered but that taxonomy does not list symptoms or symptom components, leaving this to clinicians to identify based on a person‐centred assessment of their clients. While our review does not benefit from a transdiagnostic taxonomy, we are confident that the search terms used to identify mental disorders contain the core transdiagnostic features of psychological problems, thus allowing us to capture both disorder and problems in the same search syntax (e.g., ‘anxiety’ will capture both Anxiety Disorder and anxiety as a transdiagnostic symptom). For additional cover, however, we include the following terms, and which are based on our review of some of the transdiagnostic features of mental health difficulties present in public health guidance (e.g., Health Direct Australia, 2021):

- Worried/Afraid
- Unhappy/Sad
- Emotional
- Quiet/Withdrawn
- Guilty/Worthless
- Suicide
- Suicidal Behaviour
- Self‐harm
- Mood
- Affective
- Addiction
  In some studies it may be that the level of psychological problems are measured using validated measures of distress (e.g., the Depression, Anxiety and Stress Scale [DASS‐21]; Lovibond & Lovibond, 1995). Where this is the case, then the presence of psychological problems will only be interpreted as present where the score exceeds a screening threshold that is suggestive of clinical distress. If a study treats mental health difficulties dimensionally, without the use of measures with a valid cut‐off, it will be excluded from the synthesis. However, if treated dimensionally and where dimensional data is reported for those above the cut‐off, then that data (i.e., data for those above the threshold) will be meta‐analysed.
  Psychological problems may also be identified as being present by mental health professionals, including through structured clinical interview.
  Given that the presence of disorder or problems may be assessed at different phases of terrorist involvement (e.g., before involvement, during involvement or after exiting), we will group data as follows:
  1. Where the presence‐absence of disorder or problems is assessed while the individual is involved in terrorism, or incarcerated for terrorism offences, these studies will be grouped together and referred to as ‘studies of those involved’.
  2. Where the presence‐absence is assessed after the individual has exited from terrorism, the studies will be grouped together and referred to as ‘studies of those who have exited'.
  3. Where the presence‐absence of disorder or problems is assessed as being present before the individual becoming involved in terrorism, then all studies will be grouped together and referred to as ‘disorder and problems before involvement’. For studies based on open‐source data, and where individuals in the data set came to the attention of researchers through their arrest, detention and prosecution, then the date of the index offence will be taken as the point at which the individual become involved in terrorism. Where multiple pre‐involvement time points are taken for included studies, we will group and analyse studies by the developmental periods either by separate meta‐analyses or explore through subgroup analyses using developmental period as the moderator.

To provide further context for our synthesis and discussion of findings, we will also refer to national and global estimates of point and period prevalence rates of disorder and psychological difficulties in the synthesis and discussion of findings.

### 3.2 Search methods for identification of studies

Our search strategy aligns with Cochrane Training and our past reviews for both the Cochrane Collaboration (Doody et al., 2019) and the Campbell Collaboration (Carthy et al., 2020). It is also informed by Kugley and colleagues' guidance on information retrieval for Campbell systematic reviews (Kugley et al., 2017).

#### 3.2.1 Electronic searches

We (S. C. and K. C.) will search the existing literature through the electronic platforms and databases listed in Table 2.

**Table 2:** Search platforms and databases

Platform	Database
ProQuest	International Bibliography of the Social Sciences
ProQuest	Dissertations and Theses Global
ProQuest	Social Sciences Premium
OVID	PsycArticles
OVID	PsycExtra (grey literature)
OVID	PsycInfo
Embase.com	Embase
ISI Web of Science	Web of Science Core Collection^a^
	Social Sciences Citation Index (SSCI)—1956‐present Conference Proceedings Citation Index‐ Social Science & Humanities (CPCI‐SSH)—1996‐present Book Citation Index—Social Sciences & Humanities (BKCI‐SSH)—2005‐present
Scopus	
PubMed	
OpenGrey	
EBSCO	Criminal Justice Abstracts

a Note that Chemical Indexes, Current Chemical Reactions (CCR‐Expanded), Index Chemicus (IC) and Emerging Sources Citation Index (ESCI) are included in the institutional WoS Core Collection but will be excluded from our search.

These platforms and databases have been selected as they provide coverage of journal articles across a range of publishers and disciplines, as well as indexing unpublished grey literature and academic theses.

A search syntax will be tailored for each search engine, with a preference for searches based on the title, abstract, keywords, and subject indexing fields. An example search, based on Scopus is as follows: TITLE‐ABS‐KEY (mental* OR disorder* OR illness* OR ill OR psycho* OR symptom* OR worr* OR afraid OR fear* OR unhapp* OR sad* OR emotion* OR quiet* OR withdraw* OR guilt* OR worthless* OR suicid* or *harm* OR mood* OR affect* OR addict* OR neuro* OR intellect* OR communication OR autis* OR ASD OR attention OR ADHD OR motor OR schizo* OR bipolar OR bi‐polar OR depress* OR learning OR phobi* OR anxi* OR panic OR OCD OR obsessive OR *trauma* OR post‐traumatic OR PTSD OR attach* OR stress* OR dissociat* OR somatic OR eating OR anorexi* OR sleep* OR insomni* OR compulsive OR dysphori* OR oppositional OR conduct OR antisocial OR substance* OR drug* OR alcohol* OR cognitive OR personality) AND TITLE‐ABS‐KEY (terror* OR extrem* OR radical* OR ‘political violence’ OR ‘lone actor*’ OR lone‐actor* OR Jihadi OR Salafi OR ‘right wing’ OR right‐wing OR separatist* OR nationalist* OR religious* OR guerrilla* OR paramilitary) AND TITLE‐ABS‐KEY (case‐control or ‘case control’ or correlation or coefficient* or cohort* or covariat* or cross‐section* or ‘cross section*’ or empirical or longitudinal or multivariate or odds or paramet* or predict* or prevalen* or prospective or questionnaire* or quantitative or rate* or regression or retrospective or risk* or survey* or sampl* or ‘standard deviation*’ or statistic* or variable* or variance).

Titles and abstracts for all records captured in our search will be exported into EndNote for de‐duplication and the imported into *DistillerSR* reference management software for screening.

##### 3.2.1.1 Searching other resources

It is anticipated that some relevant studies may be published as government reports or outputs from think‐tanks or other nongovernmental organisations. As such, they may not be indexed on electronic databases. To ensure these studies are identified, we (KS) will search the websites set out in Table 3.

**Table 3:** Grey literature searching

Organisation	Website
Radicalisation Awareness Network (EU)	https://ec.europa.eu/home-affairs/what-we-do/networks/radicalisation_awareness_network_en
Department of Homeland Security (US)	https://www.dhs.gov
Centre for Counter‐Terrorism Coordination (Australia)	https://www.homeaffairs.gov.au/about-us/our-portfolios/national-security/countering-extremism-and-terrorism/centre-for-counter-terrorism-coordination
Public Safety Canada	https://www.publicsafety.gc.ca/index-en.aspx
Home Office (UK).	https://www.gov.uk/government/organisations/home-office
Global Terrorism Research Centre	https://www.monash.edu/arts/social-sciences/gtrec
National Consortium for the Study of Terrorism and Responses to Terrorism (START)	https://www.start.umd.edu
Terrorism Research Centre	http://www.terrorism.org/
Hedayah	https://www.hedayahcenter.org

The Titles and Abstracts/Executive Summaries for all papers identified from this search will be identified in this process as ‘Grey Literature’ for reporting in our PRISMA chart.

We (KS) will then directly contact leading experts and expert networks (the Global Research Network on Terrorism and Technology, the European Expert Network on Terrorism issues (EENeT), VOX‐Pol Network of Excellence (NoE), the Radicalisation Awareness Network (RAN) and the Five Country Research and Development (5RD) terrorism network). Experts will be advised of the review objectives as well as the specific type of literature sought for the synthesis. Titles, abstracts and full‐texts of papers identified from these sources will be retrieved, imported into DistillerSR and placed in a folder titled ‘Experts’.

There can be a delay in indexing newly published journal articles. For this reason we (KS) will conduct a hand‐search of the following journals for papers published since January 2020:

- *Behavioural Sciences of Terrorism and Political Aggression*
- *Critical Studies on Terrorism*
- *Dynamics of Asymmetric Conflict*
- *Intelligence and Counter‐Intelligence*
- *International Journal of Conflict and Violence*
- *International Journal of Terrorism and Political Hotspots*
- *Journal of Deradicalization*
- *Journal of Policing, Intelligence and Counter‐Terrorism*
- *Journal of Terrorism Research*
- *Journal of Terrorism Studies*
- *Perspectives on Terrorism*
- *Science of Terrorism and Political Aggression*
- *Studies in Conflict and Terrorism*
- *Terrorism and Political Violence*

We (KS) will also review past systematic reviews in the area to identify papers relevant to our review (e.g., Gill et al., 2021). Citations and abstracts will be identified as ‘Hand Search’ records for reporting in our PRISMA chart.

The bibliography sections of the papers included in the review will be examined for literature that may meet our inclusion criteria (i.e., reverse citation chaining). We (K. S., S. C. and K. C.) will also identify papers that cite these relevant articles and reports using the ‘citing articles' function, where present, on search engines (i.e., forward citation chaining; Cribbin, 2011). For example, where a paper that meets our inclusion criteria is indexed on SCOPUS, a ‘Citing articles’ ribbon on the website will identify any publications indexed on the database that have cited the target article. We will also utilise Google Scholar for forward citation chaining. Articles identified through citation chaining will be recorded as ‘Chaining’ records in our PRISMA chart.

### 3.3 Data collection and analysis

#### 3.3.1 Description of methods used in primary research

Weenink (2015), for example, conducted a retrospective cohort study into the prevalence rates of mental disorder among radical Islamists from the Netherlands. Data derived from an audit of 140 cases of individuals who travelled from the Netherlands to Syria (i.e., ‘foreign fighters’) from a ‘List of Travellers’ maintained by the Counterterrorism and Extremism (CTE) team of the Dutch National Police. Weenink then reviewed their life histories by accessing several police databases. Information on mental disorder (diagnosed), psychological problems and problem behaviours were drawn from these sources. In the study, the author reports the percentage of the sample who had a diagnosed mental disorder before travelling and such information is relevant to our objectives that seeks to synthesise the literature on prevalence rates and temporality.

#### 3.3.2 Criteria for determination of independent findings

It is anticipated that multiple papers will report findings from the same study (i.e., papers reporting secondary data analysis). We will be sensitive to potentially dependent studies where the same author(s) have published the papers, they derive from the same data source in one jurisdiction (e.g., a police data set), the reported methodologies and samples are similar, and where they have been funded by the same funding source.

Where we identify two or more papers that report findings from the same data set, the papers will be treated as a single study and the team will consult to decide if (a) the study using the largest sample size should be used only or (b) we should extract data from multiple papers to generate effect sizes. In the case of the latter, for example, if one paper reported prevalence rates for anxiety and another for depression, then effect sizes could be extracted for both papers (sharing the one underlying data set).

Where there are multiple measures of the link between any mental health difficulty and the outcome (e.g., multiple measurements of anxiety), then we will use Robust Variance Estimation (RVE) to produce effect sizes, standard errors and confidence intervals (Hedges et al., 2010).

#### 3.3.3 Selection of studies

##### 3.3.3.1 Overview

The screening process for the review will be common for all objectives, commencing with title and abstract screening and followed by a full‐text review for eligibility assessment for the syntheses relating to (a) prevalence, (b) temporality, or (c) risk factor. Studies can be included in more than one of the syntheses. Details on the screening and study selection process are provided below. Two reviewers will conduct the screening, working independently, and using DistillerSR. In making decisions as to which studies are relevant to each objective, we will adopt the following rules:

1. For Objective 1 (Prevalence) we are interested in prevalence rates of mental health difficulties in terrorist populations. If the study measures prevalence of difficulties in a terrorist sample, and the design is cross‐sectional, cohort, or case‐control, and prevalence data can be drawn from the study, then we will review the paper for Objective 1.
2. For Objective 2 (Temporality) we are seeking studies that allow us to establish the prevalence rates of difficulties and where these pre‐date involvement in terrorism.
3. For Objective 3 (Risk Factor) we are interested in studies that compare the mental health difficulties of those involved and not involved in terrorism, including studies that are and are not sensitive to temporal sequencing.

##### 3.3.3.2 Title and abstract screening

Two reviewers (S. C. and K. C.) will screen records captured by the search (after de‐duplication). At this stage we will exclude documents based on the following exclusion criteria:

1. Is the paper unique (i.e., not a duplicate of another paper already in the library that was not identified using the automated function)? (Yes, No)
2. Is the paper an eligible document type (e.g., not a book review)? (Yes, No)
3. Does the study focus on mental health and terrorist behaviour? (Yes, No, Unclear)

Records with an answer of ‘No’ to any of the above criteria will be excluded. Upon completion of this phase of the screening, the reviewers will use the consistency checking function in *DistillerSR* to identify any false negative decisions. Any discrepancies will be resolved through discussion, with the involvement of a third reviewer (K. S.) if necessary.

##### 3.3.3.3 Full‐text screening

Records (documents) retained following title and abstract screening will be subject to a full‐text review in *DistillerSR*. Review authors will review each record excluding documents based on the criteria listed in Table 4. Documents included by one review author will not be examined by another author for inclusion at this stage, however, documents excluded by a review author will need to be screened independently by another review author (a screening setting in *DistillerSR*). Where a study is judged as unclear, or where there is disagreement about overall inclusion and/or which review objective the study belongs to, two study authors will resolve final inclusion decisions by discussion, involving a third author when required.

**Table 4:** Full‐text eligibility screening

*Eligibility general*		
1	Is the record unique (i.e., not a duplicate)?	1‐ No (exclude) 2‐ Yes
	*Documents which match another title exactly (i.e., title, author, year, and document type) should be excluded*.	
2	Is the document published in English?	1‐ No (excluded) 2‐ Yes
	*Document not published in English should be excluded*.	
3	Does the document report the results of an empirical study?	1‐ No (excluded) 2‐ Yes
	*Documents that do not report empirical findings (e.g., conceptual papers, newspaper articles and minutes of meetings) should be excluded*.	
6	Does the study include a sample (or subsample) of individuals involved in terrorism as defined in the protocol?	1‐ No (excluded) 2‐ Yes (move to next question)
*Eligibility Objective 1 Prevalence*		
7	Does the study measure the presence of mental health difficulties (as defined in the protocol) in the terrorist sample or subsample?	1‐ No (excluded) 2‐ Yes (included for Objective 1, move to next question to consider Objective 2 and 3)
*Eligibility Objective 2 Temporality*		
8	Does the study establish that the mental health difficulties preceded the terrorist behaviour?	1‐ No (excluded for Objective 2) 2‐ Yes (included for Objective 2, move onto next question to consider eligibility for Objective 3)
*Eligibility Objective 3 Risk Factor*		
9	Does the study include a sample where some individuals were involved in terrorism and others were not?	1‐ No (excluded for Objective 3) 2‐ Yes (move to next question)
10	Does the study measure the presence of mental health difficulties, for both groups, as defined in the protocol?	1‐ No (excluded for Objective 3) 2‐ Yes (move to next question)
11	Does the study report the relationship between the absence/presence of mental health difficulties and involvement in terrorism (involved/not involved), or provide sufficient information to calculate that relationship?	1‐ No (excluded for Objective 3) 2‐ Yes

Cohen's Kappa (*κ*) will be calculated for testing inter‐rater reliability between both screeners' study selections. Although the kappa statistic will be used to guide the initial assessment of the level of inter‐rater agreement, in line with Cochrane Review recommendations (e.g., Li et al., 2022), the focus will be on reasons for disagreements (e.g., reviewer bias) rather than the kappa statistic itself. Disagreements between S. C. and K. C. will be resolved through discussion with a third reviewer (K. S.). The third reviewer (K. S.) will also search for errata to included studies, and where present reassess the eligibility of each study based on the inclusion/exclusion criteria.

#### 3.3.4 Data extraction and management

For each study, we will extract key information including study authors, design, population (e.g., convicted offenders etc.), data source, data type, diagnostic approach, diagnoses, symptoms, ‘type’ of terrorism, ‘role’ of terrorist, comparison group, and summary results. This data will be recorded in detailed data extraction tables (see Supporting Information: Appendix A: Full‐Text Coding Form).

#### 3.3.5 Dealing with missing data

Where data needed for our analysis are missing from a paper, we will contact the corresponding author, or other authors where the corresponding author does not respond, seeking access to additional data. Where this is not available then that paper will be excluded from the meta‐analysis but retained for the narrative synthesis.

#### 3.3.6 Assessment of risk of bias in included studies

Based on our pilot screening and knowledge of the wider literature, we anticipate that the majority of papers that meet our inclusion criteria will report prevalence data for terrorist samples, or for both terrorist and non‐terrorist samples (i.e. case control designs). For that reason, we will use the Joanna Briggs Institute Quality Assessment Checklist for Prevalence Data for all studies under Objective 1 and 2, and then augment the checklist with the JBI Checklist for Case Control Studies for studies included under Objective 3. Items are presented in the Full‐Text Coding Form at Appendix A and assessments will be informed by explanatory guides for the tools (e.g., Munn et al., 2014).

The risk of bias for each study will be interpreted as follows:

- Low risk of bias: All items are rated as ‘Yes’
- High risk of bias: At least one item rated as ‘No’.
- Unclear risk of bias: One item is listed as ‘unclear’ and the remainder as ‘Yes’.

We have added an additional Risk of Bias item to the JBI checklists—one that is sensitive to plausibility as discussed earlier in this protocol. Where studies present a theoretical argument linking mental health difficulties with terrorist involvement the quality criterion will be indicated as having been met. Our Risk of Bias discussion will consider these theoretical arguments with regard to the 4Ps.

The risk of bias assessment will be conducted by two independent coders and reliability will be tested using the Kappa statistic *κ*. In line with Campbell Collaboration practice and policies, studies will not be excluded based on their risk of bias or quality. Instead, all studies for which effect sizes can be obtained will be included in the meta‐analyses. In reporting the results of the risk of bias assessment, we will do so by clustering studies by design and reporting the ratings for each domain across all studies assessed in tables, which will be accompanied by a written summary and rationale for our ratings.

#### 3.3.7 Assessment of publication bias

Negative results are less likely to be published and/or made broadly available for syntheses of this nature (Joober et al., 2012). As such, there may be an increased risk of reporting biases in the identified literature. To determine if the synthesised data is subject to such a publication bias, a contour enhanced funnel plot (Palmer et al., 2008) Trim and Fill test (Duval, 2005) and Egger's regression test (Egger et al., 1997) will be used.

### 3.4 Data synthesis

Data synthesis will be completed using Comprehensive Meta‐Analysis (CMA) software (Borenstein et al., 2013).

For Objective 1 (Prevalence), proportion data (i.e., proportion of the terrorist sample with a mental health difficulty) will be transformed into a logit for performing the meta‐analysis. The meta‐analysis will assume a random‐effects model a priori and will use the REML estimator of the random‐effects variance component. Mean effect sizes and associated 95% confidence intervals will be back‐transformed into proportions (prevalence) for ease of interpretation.

For Objective 1 we will also draw on published national and global (e.g., World Health Organization World Mental Health Surveys) point (e.g., at time of survey/assessment) and period (e.g., life‐time, last 12‐months etc.) prevalence rates of mental disorder and psychological difficulties to provide a benchmark against which the prevalence rates can be assessed. There is likely to be multiple estimates available and these will be tabulated and ranges of estimates identified as reference points.

For Objective 2 (Temporality) we will replicate this analysis including only those papers where authors assert that the mental health difficulties pre‐dated involvement in terrorism.

Finally for Objective 3 (Risk Factor) the effect size will be the odds ratio between mental health diagnosis and later involvement in terrorism. Again, the meta‐analysis will assume a random effects model a priori and use the REML estimator of the random effects variance component.

It may be the case that multiple studies report binary data for mental health difficulties (disorder present/absent) based on scores on a screening tool and then present continuous data for those scoring above the threshold/cut‐off. In such an event, we will use Cohen's *d* as the measure of effect size. We will convert these to odds ratios using the logit method. However, we will also analyse these separately to assess whether the using a threshold/cut‐off or continuous measure of mental health affects the observed effect size.

In each analysis we will deal with the diversity of mental health problems by grouping effect sizes into related categories of problems (e.g., depression, anxiety etc.) for meta‐analyses. We will run separate analyses for each category along with a corresponding forest plots.

#### 3.4.1 Subgroup analysis and investigation of heterogeneity

Heterogeneity will be explored statistically using the homogeneity *Q*‐statistic and the *I* ^2^ test. Tau squared will be reported along with each mean effect size. In line with the Cochrane Handbook (Deeks et al., 2019) if the meta‐analysis contains at least ten studies, it may be possible to conduct further sub‐group analysis (see also Richardson et al., 2019). Although ten studies may not be sufficient (i.e., if the covariate is unevenly distributed), it is predicted that the included studies will have large sample sizes, and ten studies will be sufficient to produce useful findings in the sub‐group analysis.

At this stage we envisage that it may be possible to conduct moderator analysis based on ‘Type’ of terrorism (Lone actor; Islamic; Right‐Wing; Separatist; Mixed; Other), onset of disorder/problems relative to becoming involved in terrorism (pre, during, post, mixed, not‐clear), developmental age of onset of difficulties (e.g., child/adolescent <18 vs. adult 18+), developmental age of involvement in terrorism (e.g., e.g., child/adolescent <18 vs. adult 18+), type of design (retrospective vs prospective) and the time period of interest (e.g., last 5‐years, 2‐years, 12 months etc).

The coding categories detailed in Appendix A will likely serve as suitable moderators to formally explore the mean effect across sub‐groups (e.g., ‘type’ of terrorism or ‘role’ of terrorist). This will be conducted using one‐way random effects analog‐to‐the ANOVA, with separate variance estimates within sub‐groups. That is, the differences across subgroups will be assessed using the *Q* statistic directly analogous to an *F* test if there are three or more means or a *t* test if there are two means. Additionally, each subgroup will have a unique mean, confidence interval, and random effects variance component.

As part of the sub‐group analysis we will consider how terrorist behaviour was measured within the studies included. Our coding form identifies five sources of information that is often used to determine whether or not an individual has engaged in terrorist behaviour (police database, self‐report, case notes, media reports or court reports), and we have included an ‘other' category for studies that use mixed sources or other sources.

### 3.5 Sensitivity analysis

As mentioned, studies will not be excluded based on their risk of bias but will undergo additional analyses in the form of sensitivity analyses. Sub‐groups of studies will be selected based on bias risk and all analyses will be re‐run without those posing a ‘high’ risk of bias. The purpose of this additional step is to formally explore if the observed effect sizes are dependent on the inclusion of studies.

### ROLES AND RESPONSIBILITIES

Dr. Kiran Sarma is an expert in the psychology of risky and extreme behaviour, including political violence and terrorism. Dr Sarma also has extensive experience in systematic review methods and information retrieval, having co‐authored several systematic reviews and meta‐analyses on the topics of risk taking.

Sarah Carthy has completed a PhD in Psychology with a focus on violent radicalisation. Having authored a systematic review with the Campbell Collaboration on counter‐narratives, she has a good knowledge of systematic review methods, statistical analysis and information retrieval.

Katie Cox is a forensic psychologist. She has a degree in Psychology, and expertise in informational retrieval and systematic review methods. She is also a co‐author on the counter‐narratives systematic review mentioned above.

The specific contributions are as follows:

- Content: K. S will lead, S. C. and K. C. will support.
- Systematic review methods: K. S. will manage, and S. C. and K. C. will execute the search and extraction.
- Statistical analysis: K. S. will lead, S. C. will support.
- Information retrieval: S. C. and K. C. will lead and K. S. will support and audit.

## CONFLICT OF INTEREST

The authors declare no conflict of interest.

## PRELIMINARY TIMEFRAME

The search and retrieval process will conclude by May 2022 with submission for peer‐review by June 2022.

## Supporting information

Supporting information.
