# Supplementary material for: PROTOCOL: Mental disorder, psychological problems and terrorist behaviour: A systematic review
Source: Campbell Syst Rev. 2022 Jun 10;18(2):e1249. doi: 10.1002/cl2.1249 (PMC9186052; doi:10.1002/cl2.1249)
Supplement: Supplementary file 1 — Supporting information. [file CL2-18-e1249-s001.docx]

**APPENDIX A. Full-Text Coding Form**

1. **General Study Details**

| Field | Response Options | TYPE |
| --- | --- | --- |
| Document ID |  | Numeric |
| Coder | - - - 1. SC       2. KC       3. KS | Categorical |
| Date Coded |  | Numeric |
| Study Author |  | Text |
| Study Title |  | Text |
| Publication Type | 1. Book  2. Book Chapter  3. Journal Article (peer reviewed)  4. Government Report (not police/security)  5. Police/Security Service Report  6. Academic Thesis Doctoral Level  7. Academic Thesis Other Level  8. Report of Expert Network  9. Conference Paper  10. Other | Categorical |
| Publication Other Specify |  | Text |
| Journal Name |  | Text |
| Journal Volume |  | Numeric |
| Journal Issue |  | Numeric |
| Jurisdiction Data Gathered |  | Text |
| Date Range Data Gathered | Commence; Finish | Numeric |
| Funding Source |  | Text |
| Data Source | - - - 1. Police Database       2. Self-Report Questionnaire       3. Case Notes       4. Media Reports       5. Court Reports       6. Other | Categorical |
| Data Source Other Specifcy |  | Text |
| Data reported in other study | 1.Yes  2.No | Categorical |
| Data reported in other study Yes | Author, Year | Text |

1. **Sample**

| Field | Response Options | TYPE |
| --- | --- | --- |
| Population Size |  | Numeric |
| Description of Sample | 1. Prisoners  2. Former Terrorists  3. Active Terrorists  4. Other | Categorical |
| Description of Sample Other |  | Text |
| Gender | - - - 1. Male       2. Female       3. Both       4. Other | Categorical |
| Gender Other Specify |  | Text |
| Gender breakdown | % Male, % Female, % Other | Text |
| Ethnicity |  | Text |
| Age | Mean; SD; Range | Numerical |
| Developmental age | 1. 0-17 Child/Adolescent  2. 18+ Adult |  |

**C.Outcome**

| Field | Response Options | TYPE |
| --- | --- | --- |
| Definition of Terrorism |  | Text |
| Type of Terrorism | Lone actor  Islamic  Right Wing   - - - 1. Separatist       2. Mixed       3. Other | Categorical |
| Type Mixed Specify |  | Text |
| Type Lone Actor Specify |  | Text |
| Type Other Specify |  | Text |
| Role | (e.g., bombing; logistics; finance; propaganda; unknown; mixed). | Text |

**D.Risk Factor**

| Field | Response Options | TYPE |
| --- | --- | --- |
| Diagnosed Mental Disorders | Yes  No  Unclear | Categorical |
| Yes Disorders Specify | 1. Neurodevelopmental Disorder  2. Intellectual Disability  3. Communication Disorder  4. Autism Spectrum Disorder  5. Attention-Deficit/Hyperactivity Disorder  6. Specific Learning Disorder  7. Motor Disorder  8. Other Neurodevelopmental Disorder  9. Schizophrenia or Psychotic Disorder  10. Bipolar and Related Disorder  11. Depressive Disorder  12. Anxiety Disorder  13. Obsessive-Compulsive and Related Disorders  14. Trauma and Stressor-Related Disorders  15. Dissociative Disorder  16. Somatic Symptom and Related Disorders  17. Feeding and Eating Disorders  18. Elimination Disorders  19. Sleep-Wake Disorders  20. Gender Dysphoria  21. Disruptive, Impulse-Control and Conduct Disorders  22. Substance-Related and Addictive Disorders  23. Neurocognitive Disorders  24. Cluster A Personality Disorder  25. Cluster B Personality Disorder  26. Cluster C Personality Disorder  27. Other Personality Disorder  28. Paraphilic Disorders  29. Other | Categorical (select all that apply) |
| Other Specify |  | Text |
| Diagnosed by mental health professional | 1. Yes  2. No | Categorical |
| Diagnosed by prof. Yes Describe | e.g., Clinical interview, case note review, testing | Text |
| Diagnosed by prof No Describe | Justify inclusion as disorder | Text |
| Co-Morbidity |  | Text |
| Psychological Problems | 1. Worried/Afraid  2. Unhappy/Sad  3. Emotional  4. Quiet/Withdrawn  5. Guilty/Worthless  6. Suicide  7. Suicidal Behaviour  8. Self-harm  9. Mood  10. Affective  11. Addiction  12. Other | Categorical (select all that apply) |
| Problems Other Specify |  | Text |
| Problems vs. Clinical Psychological Problems? |  | Text |
| How problems determined in study | - - - 1. 1.Open Source       2. 2.Offical Data (including police reports; court reports etc.)   3.Family/Peer reports   - - - 1. 4.Mental Health records   5.Other | Categorical (select all that apply) |
| Problems determined other specify |  | Text |
| Onset of disorder relative to terrorism involvement | 1.Pre  2.During  3.Post  4.Not clear  5.Mixed |  |
| Onset Disorder not clear or mixed Specify |  | Text |
| Onset of problems relative to terrorism involvement | 1.Pre  2.During  3.Post  4.Not clear  5.Mixed | Categorical |
| Onset problems not clear or mixed Specify |  | Text |

**E. Study Design**

| Field | Response Options | TYPE |
| --- | --- | --- |
| Design | 1.Cross-Sectional  2.Cohort Retrospective  3.Cohort Prospective  4.Case-Control  5.Other | Categorical |
| Design Description |  | Text |
| Design Other |  | Text |
| Relevant Objective 1. | 1. Study estimates the proportion of sample terrorist population that has a mental health difficulty/disorder (i.e., Include in Objective 1). | Categorical |
| Relevant Objective 2. | 1. Study estimates the association between mental health difficulties disorder and terrorist behaviour (i.e., Include in Objective 2). | Categorical |
| Time Points Retrospective |  | Text |
| Time Points Prospective |  | Text |
| Control Sample Type |  | Text |
| Control Sample Size |  | Text |
| Control Sample Data Source |  | Text |
| Control Sample Data Type | (e.g., prevalence, point-prevalence etc.) | Text |
| Control Sample Diagnostic Approach | (e.g., Clinical interview) | Text |
| Control Sample diagnosis(es) |  | Text |
| Control Sample problems |  | Text |
| Control Sample Temporal onset | (e.g., prior to incarceration; childhood etc.) | Text |
| Control Sample duration of disorder |  | Text |
| Control Sample duration of problems |  | Text |

F. Findings

| Field | Response Options | TYPE |
| --- | --- | --- |
| If case control, was difference significant | 1.Yes  2.No  3.Unclear  4.Not tested | Categorical |
| If multiple tests, describe all |  | Text |
| If case control, was standardised effect size reported? | 1.Yes  2.No | Categorical |
| Significance Tests reported | 1.t-value  2.F-value  3. Chi-Square value |  |
| If multiple tests, describe all |  | Text |
| If ‘Yes’ case control effect what measure | 1.r  2.b  3.OR  4.Other | Categorical |
| Effect size | (e.g., Test 1; Test 2; Test 3 etc.) | Numerical |
| Standard error of effect | (e.g., SE1; SE2; SE3 etc.) | Numerical |
| Page number(s) effects reported |  | Numerical |
| If no effect, does data allow effects to be calculated? | 1.Yes  2.No | Numerical |
| Point Prevalence Rate Reported for all Disorders + Confidence Intervals |  | Numerical |
| Point Prevalence Rate Reported for specific Disorders + Confidence Intervals |  | Numerical |
| Point Prevalence Rate Reported for all Problems + Confidence Intervals |  | Numerical |
| Point Prevalence Rate Reported for specific Problems + Confidence Intervals |  | Numerical |
| Period Prevalence Rate Reported for all Disorders + Confidence Intervals |  | Numerical |
| Period Prevalence Rate Reported for specific Disorders + Confidence Intervals |  | Numerical |
| Period Prevalence Rate Reported for all Problems + Confidence Intervals |  | Numerical |
| Period Prevalence Rate Reported for specific Problems + Confidence Intervals |  | Numerical |
| Plausibility arguments | 1.Presents theoretical link  2.Does not present theoretical link  3.Unclear |  |
| Plausibility arguments describe | Summarise author(s)’ argument on link between mental health difficulties and terrorism with reference to a) predisposing, b) precipitating, c) perpetuating and d) protective processes. | Text |
| Findings describe | Summarise key findings | Text |
| Author(s)’ conclusions | 1.Mental health difficulties confers risk  2.Mental health difficulties does not confer risk  3.Mental heath difficulties reduces risk  4.Evidence insufficient  5.No conclusions | Categorical |
| Author(s)’ conclusions expanded | Summarise key conclusions | Text |

**G. Risk of Bias (Prevalence)**

| Field | Response Options | TYPE |
| --- | --- | --- |
| Was the sample frame appropriate to address the target population? | 1.Yes  2.No  3.Unclear  4.Not Applicable | Categorical |
| Were study participants recruited in an appropriate way? | 1.Yes  2.No  3.Unclear  4.Not Applicable | Categorical |
| Was the sample size adequate? | 1.Yes  2.No  3.Unclear  4.Not Applicable | Categorical |
| Were the study subjects and the setting described in detail? | 1.Yes  2.No  3.Unclear  4.Not Applicable | Categorical |
| Was the data analysis conducted with sufficient coverage of the identified sample? | 1.Yes  2.No  3.Unclear  4.Not Applicable | Categorical |
| Were valid methods used for the identification of the condition? | 1.Yes  2.No  3.Unclear  4.Not Applicable | Categorical |
| Was the condition measured in a standard reliable way for all participants? | 1.Yes  2.No  3.Unclear  4.Not Applicable | Categorical |
| Was there appropriate statistical analysis? | 1.Yes  2.No  3.Unclear  4.Not Applicable | Categorical |
| Was the response rate adequate, and if not, was the low response rate managed appropriately | 1.Yes  2.No  3.Unclear  4.Not Applicable | Categorical |

Risk of Bias – Case Control

| Field | Response Options | TYPE |
| --- | --- | --- |
| Were the groups comparable other than the presence of disease in cases or the absence of disease in controls? | 1.Yes  2.No  3.Unclear  4.Not Applicable | Categorical |
| Were cases and controls matched appropriately? | 1.Yes  2.No  3.Unclear  4.Not Applicable | Categorical |
| Were the same criteria used for identification of cases and controls? | 1.Yes  2.No  3.Unclear  4.Not Applicable | Categorical |
| Was exposure measured in a standard, valid and reliable way? | 1.Yes  2.No  3.Unclear  4.Not Applicable | Categorical |
| Was exposure measured in the same way for cases and controls? | 1.Yes  2.No  3.Unclear  4.Not Applicable | Categorical |
| Were confounding factors identified? | 1.Yes  2.No  3.Unclear  4.Not Applicable | Categorical |
| Were strategies to deal with confounding factors stated? | 1.Yes  2.No  3.Unclear  4.Not Applicable | Categorical |
| Were outcomes assessed in a standard, valid and reliable way for cases and controls? | 1.Yes  2.No  3.Unclear  4.Not Applicable | Categorical |
| Was the exposure period of interest long enough to be meaningful? | 1.Yes  2.No  3.Unclear  4.Not Applicable | Categorical |
| Was appropriate statistical analysis used? | 1.Yes  2.No  3.Unclear  4.Not Applicable | Categorical |

Risk of Bias – Cross Sectional

| Field | Response Options | TYPE |
| --- | --- | --- |
| Were the criteria for inclusion in the sample clearly defined? | 1.Yes  2.No  3.Unclear  4.Not Applicable | Categorical |
| Were ere the study subjects and the setting described in detail? | 1.Yes  2.No  3.Unclear  4.Not Applicable | Categorical |
| Was the exposure measured in a valid and reliable way? | 1.Yes  2.No  3.Unclear  4.Not Applicable | Categorical |
| Were objective, standard criteria used for measurement of the condition? | 1.Yes  2.No  3.Unclear  4.Not Applicable | Categorical |
| Were confounding factors identified? | 1.Yes  2.No  3.Unclear  4.Not Applicable | Categorical |
| Were strategies to deal with confounding factors stated? | 1.Yes  2.No  3.Unclear  4.Not Applicable | Categorical |
| Were the outcomes measured in a valid and reliable way? | 1.Yes  2.No  3.Unclear  4.Not Applicable | Categorical |
| Was appropriate statistical analysis used? | 1.Yes  2.No  3.Unclear  4.Not Applicable | Categorical |
